# MRS of Brain Metabolite Levels Demonstrates the Ability of Scavenging of Excess Brain Glutamate to Protect against Nerve Agent Induced Seizures

**DOI:** 10.3390/ijms16023226

**Published:** 2015-02-02

**Authors:** Angela Ruban, Inbal E. Biton, Arik Markovich, David Mirelman

**Affiliations:** 1 Department of Neurobiology, Weizmann Institute of Science, Rehovot 76100, Israel; E-Mail: mr.kovich.a@gmail.com; 2 Department of Veterinary Resources, Weizmann Institute of Science, Rehovot 76100, Israel; E-Mail: inbal.biton@weizmann.ac.il; 3 Department of Biological Chemistry, Weizmann Institute of Science, Rehovot 76100, Israel; E-Mail: david.mirelman@weizmann.ac.il

**Keywords:** brain glutamate scavengers, oxaloacetate, hGOT, MRS, lactate

## Abstract

This study describes the use of *in vivo* magnetic resonance spectrocopy (MRS) to monitor brain glutamate and lactate levels in a paraoxon (PO) intoxication model. Our results show that the administration of recombinant glutamate-oxaloacetate transaminase (rGOT) in combination with oxaloacetate (OxAc) significantly reduces the brain-accumulated levels of glutamate. Previously we have shown that the treatment causes a rapid decrease of blood glutamate levels and creates a gradient between the brain and blood glutamate levels which leads to the efflux of excess brain glutamate into the blood stream thereby reducing its potential to cause neurological damage. The fact that this treatment significantly decreased the brain glutamate and lactate levels following PO intoxication suggests that it could become a new effective neuroprotective agent.

## 1. Introduction

Exposure to organophosphate nerve agents causes a set of severe symptoms, such as seizures that rapidly progress to status-epilepticus, which leads to profound structural brain damage [1]. Much of the brain damage does not typically occur at the time of the initial lesion, making secondary neurological damage a major contributor to the neuronal loss [2]. This damage is partially related to the release of excessive amounts of glutamate (Glu) into the brain interstitial/cerebrospinal fluid (ISF/CSF) [3]. Glu plays a substantial role in the propagation and maintenance of organophosphates-induced seizures, thus contributing to the secondary brain damage [4]. Furthermore, Glu receptors’ antagonists in general, and *N*-methyl-d-aspartate (NMDA) blockers in particular, were proposed as potential antidotes against organophosphates intoxication [5].

The maintenance of brain extracellular Glu at levels below its excitotoxic threshold is performed not only by Glu transporters located on glia and neurons but also by those present on the anti-luminal side of the brain capillary endothelial cells [6,7,8]. The present study is based on our previous findings that excess brain glutamate can be reduced by the application of a novel blood glutamate scavenging (BGS) treatment that rapidly decreases glutamate levels in the blood, thereby increasing the driving force for Glu fluxes from brain ISF/CSF to the blood [9]. The rapid decrease of plasma Glu levels is achieved by the intravenous administration of a recombinant preparation of glutamate oxaloacetate transaminase (rGOT) in combination with low amounts of the co-substrate oxaloacetate (OxAc). Administration of rGOT in combination with OxAc, converts Glu into 2-ketoglutarate and aspartate, thereby decreasing the blood concentration of Glu. BGS provided highly significant brain neuroprotection in rat animal models of closed head injury, ischemic stroke, glioma brain tumors and paraoxon (PO) intoxication [10,11,12,13]. In addition, it was showed that under hypoglycemic conditions, extracellular Glu can be transformed from a neurotoxin to a survival factor by GOT, provided there is sufficient oxygen to sustain cellular respiration in the rodent Stroke model [14].

Lactate is one of the main cerebral metabolites and the presence of excess lactate level in CSF indicates that glycolysis was activated in an oxygen deficient environment. Several causes for this activation have been described in many pathological conditions, such as ischemia, hypoxia, mitochondrial disorders, epilepsy and organophosphate intoxication [15,16,17]. It was shown that the brain lactate/creatine levels as measured 3 h post Paraoxon (PO) intoxication were in positive correlation with 24 h brain edema [17]. These changes were found to correlate with short-term prognosis, *i.e.*, survival.

In this study we used *in vivo* magnetic resonance spectroscopy (MRS), to determine levels of glutamate and lactate in the brains of rats before and after intoxication with PO in BGS treated and non-treated animals. The use of the high magnetic field (9.4 T) MRS allows the accurate non-invasive detection of the levels of most brain metabolites in a certain brain region. At low magnetic field strength the broad resonance centered at approximately 2.2 ppm contains overlapping resonances arising from glutamate, glutamine (Gln) and gamma-aminobutyric acid (GABA), which are often indistinguishable. To avoid confusion in spectral assignment of Glu, Gln and GABA, a term glutamix (Glx) can been used to reflect the combination of Glu and Gln concentration (*i.e*., Glx = Glu + Gln + GABA). High magnetic field strength has been used in this study and can separately resolve Glu and Gln peaks. Brain metabolite concentrations are usually expressed as ratios (relative quantification, mostly normalized to the peak of phosphocreatine) rather than as absolute concentrations [18]. The level of most common cerebral metabolites can be altered under pathological conditions or after drug administration while the level of phosphocreatine (PCr) remains mostly constant [17].

The aim of this study was to confirm the efficiency of using BGS as a novel neuroprotective treatment in PO intoxication model by real-time monitoring the reduction of the excess brain Glu levels following BGS treatment. This data could provide the proof of concept of the effectiveness of BGS as a neuroprotective treatment in the PO intoxication model.

## 2. Results and Discussion

### 2.1. Clinical Signs

**Figure 1:**
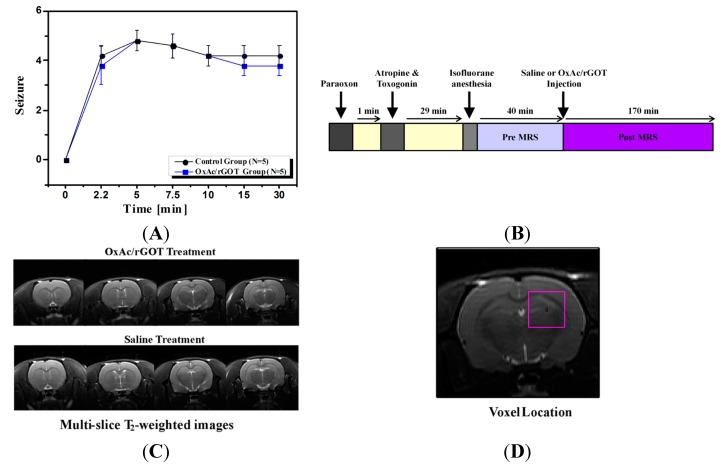
**(A)** Racine’s scale evaluation of seizure’s post paraoxon (PO) exposure; (**B**) magnetic resonance spectrocopy (MRS) experiment protocol scheme; (**C**) four axial T_2_ (spin–spin relaxation)-MR-weighted images of representative rat brains treated with saline or oxaloacetate (OxAc)/glutamate-oxaloacetate transaminase (rGOT). The field of view of the the image was 4 × 4 cm^2^; (**D**) Axial MR image of the rat brain obtained by RARE (rapid acquisition with relaxation enhancement) sequence showing the position of the selected voxel (4 × 4 × 4 mm^3^) covering predominantly the hippocampus region. The field of view of the entire image was 4 × 4 cm^2^.

In all PO-challenged animals, clinical signs characteristic of organophosphates intoxication and consisting of both peripheral and central cholinomimetic manifestations were noted. Following PO injection all the 10 rats tested developed behavioral seizures corresponding to stages 1–5 of Racine’s classifications, lasting up to the 30 min after the PO injection. The intensity and duration of the clinical signs were similar in both groups (Figure 1A).

### 2.2. Effect of Blood Glutamate Scavenging (BGS) Treatment on Brain Glutamate and Lactate Levels

**Figure 2:**
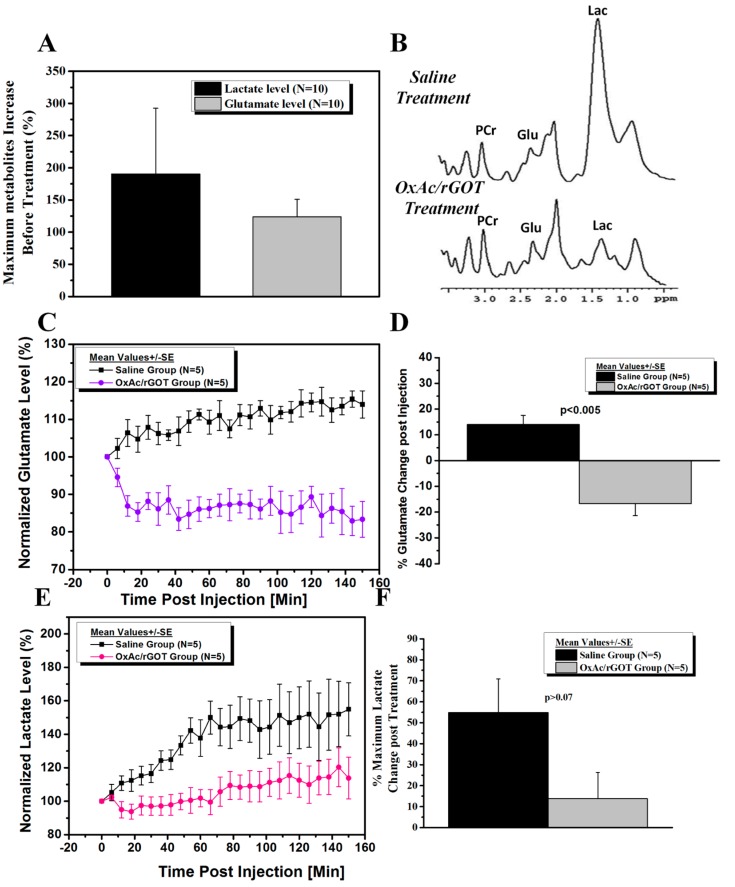
(**A**) Post PO exposure before treatment, lactate and glutamate levels increased in comparison with baseline values before the exposure to PO. Data were expressed as mean ± S.E. *p* < 0.05 for both metabolites as determined by *t*-test (in comparison with baseline values); (**B**) Representative *in vivo* proton MRS spectra in the hippocampus after OxAc/rGOT and saline treatments at the latest MRS time point. Lactate (Lac) is resolved at 1.3 ppm, glutamate (Glu) at 2.2 ppm and phosphocreatine (PCr) at 3.0 ppm; (**C**) Normalized glutamate levels in the hippocampus after OxAc/rGOT and saline treatments as a function of time. Data were expressed as mean ± S.E. *p* < 0.005 as determined by *t*-test; (**D**) The hippocampus glutamate levels after the OxAc/GOT or saline treatments at the end of the experiment; (**E**) Normalized lactate levels in the hippocampus after OxAc/rGOT and saline treatments as a function of time; (**F**) The hippocampus lactate levels after the OxAc/GOT or saline treatments at the end of the experiment. Data were expressed as mean ± S.E. *p* < 0.04 as determined by *t*-test.

Ten rats were injected with PO and treated with saline or OxAc/rGOT (Figure 1B). Axial images along with a spectroscopic voxel location in hippocampus area are shown in Figure 1D. The T_2_ (spin–spin relaxation)-weighted MR images (Figure 1C) of the two groups of rats predictably appeared similar. The T_2_-weighted MR images were acquired before the saline or OxAc/rGOT injection and also 70 min post injection. These images were similar and excluded the possibility of gross morphological or water content differences between pre and post (70 min) saline or OxAc/rGOT injection. The high-resolution localized 1H MR spectra measured after the PO exposure revealed a significant increase in the glutamate and lactate levels in the hippocampus of all animals (Figure 2A) in comparison with baseline values before the exposure to PO. Seventy minutes post PO injection the rats were treated with OxAc/rGOT or saline (five rats per each group) and the hippocampus metabolites were measured during 150 min post treatment. Figure 2B shows the 1H representative spectra in the hippocampus after OxAc/rGOT and saline treatments at the latest MRS time-point. The difference in the hippocampus metabolites after the two treatments is very clear from these spectra. In the rat-treated group that was injected with OxAc/rGOT, the brain glutamate level significantly decreased up to ~84% in the first 20 min and remained in the same level up to the end of the experiment (Figure 2C). In contrast to the treated group, in the saline group the glutamate level continuously increased up to ~115% after 150 min post-treatment (Figure 2C). At the end of the experiment, the hippocampus glutamate levels were −16.4% ± 4.7% and 14.0% ± 3.6%, in the OxAc/rGOT or saline groups, respectively (Figure 2D).

The lactate levels after both treatments continuously increased until the end of the MRS protocol (Figure 2E). The peak at 1.3 ppm is assigned to lactate and also lipid/macromolecule. Lipid peaks are detectable by short echo proton spectroscopy at 0.9 and 1.3 ppm. The lipids levels (at 0.9 ppm) were measured during the experiment at both saline and treated rat groups. The lipid level after both treatments did not changed until the end of the MRS protocol. Therefore, we assume the rise of the peak at 1.3 ppm after PO exposure is assigned to brain lactate concentration. Interestingly, we found that the OxAc/rGOT treatment also caused a significant decrease in the lactate levels in comparison to the control group especially up to 70 min post-treatment. At the end of the experiment, the hippocampus lactate levels were +13.8% ± 12.5% in the OxAc/rGOT group and 54.9% ± 15.9%, in the saline group (Figure 2F).

It is important to mention that the measurements of glutamate started 30 min post PO injection, which means that the glutamate and lactate levels were already at high levels as a result of the seizures. In the preliminary experiments we found that before PO injections, the animals displayed variable low baseline levels of glutamate. However, since all brain metabolite levels measurements have to be done in the Magnetic Resonance Imaging (MRI) in anesthetized animals, we found that it was not accurate to correlate the baseline measurements of glutamate in each animal with those obtained after the PO exposure if performed after two subsequent anesthetic regimens (one before and one after the PO exposure). Therefore, glutamate and lactate levels were calculated for each animal as a percentage of the changes observed from the first measurement following the injection of either saline or BGS.

The results obtained provide a direct proof for the ability of the BGS treatment to rapidly reduce excess brain Glu and lactate levels for an extended period.

## 3. Experimental Section

### 3.1. Materials

Paraoxon was obtained from Chem Service (West Chester, PA, USA), all other materials were obtained from Sigma-Aldrich (St. Louis, MO, USA). Recombinant His-tagged version of the human glutamate oxaloacetate transaminase (rGOT) cDNA, that was cloned from the human hepatoma cell line hepG2, was expressed in BL21(DE3) *E. coli* cells using isopropyl β-d-1-thiogalactopyranoside (IPTG). After protein expression cells were harvested and lysed using sonication. The soluble fraction of *E. coli* extract was recovered by centrifugation, and human glutamate oxaloacetate transaminase 1 (GOT1) protein was purified on Nickel-Nitrilotriacetic Acid resin (Ni-NTA) affinity column. The purified protein was then concentrated using a Viviaspin (membrane cutoff 30 KDa), and by exchanging the buffer to PBS supplemented with 1 mM pyridoxal phosphate and 1 mM a-ketoglutarate. Finally, the quantity and quality of the purified GOT1 was determined by running the samples on an SDS-PAGE gel, and by measuring the protein absorbance at 280 nm, and estimating its concentration using the extinction coefficient. The enzyme was produced by Dr. Ghil Yona in Department of Biological services, Weizmann Institute of Science, Rehovot, Israel.

### 3.2. Animals

The experiments were conducted according to the Guidelines for the Use of Experimental Animals of the European Community approved by the Animal Care Committees of the Weizmann Institute of Science, under permit number of 09040214-2 (decision date: 25 February 2014). Ten healthy male Sprague–Dawley rats, 8–9 weeks old 260–270 gram, were used for the main study and also four rats for the preliminary experiments.

### 3.3. Study Design

At the day of the experiment PO was injected intramuscular (IM) at the hind limb with a dosing of 450 µg/kg. One-minute post organophosphates challenge, Atropine and Toxogonin at a dose of 0.9 mg/100 µL and 6 mg/100 µL per animal respectively were administered IM as well. The rats were randomly divided into two groups. Intravenous (IV) infusion of the OxAc/rGOT treatment solution or 0.9% saline as a control were carried out with the aid of a tail vein cannula inserted 5 min prior to the respective treatment. Subsequently and starting at 30 min post PO challenge, infusion of the OxAc/rGOT or 0.9% saline were initiated and administered at a dose of OxAc 4.5 µg/animal and rGOT 45 µg/animal in a final volume of 2 mL/animal by push injection (Figure 1B). During the MRI scanning, rats were anesthetized with isofluorane (5% for induction, 1%–2% for maintenance) mixed with oxygen (1 L/min) and delivered through a nasal mask. Once anesthetized, the animals were placed in a head-holder to assure reproducible positioning inside the magnet. Respiration rate was monitored by a pressure sensor placed under the abdomen of the animals (SA Instruments, Inc, New York, NY, USA) and kept throughout the experimental period around 60–80 breaths per min. Body temperature of the animals was also controlled and kept at 37 °C using a warm water blanket. At the end of the study all the animals were sacrificed.

### 3.4. Clinical Signs

All animals were observed for clinical signs with particular attention devoted to the onset, intensity and duration of characteristic and representative peripheral and central cholinomimetic manifestations. The latency until evident onset of convulsing seizures, as well as the intensity, scored by use of the Racine’s scale and respective time of cessation was documented. Clinical signs observations were carried out prior to PO administration and thereafter at 1/2, 5/2, 5, 15/2, 10, 15, 30 min post-dosing.

### 3.5. Magnetic Resonance Imaging (MRI)

MRI experiments were performed on 9.4 Tesla BioSpec Magnet 94/20 USR system (Bruker BioSpin, Ettlingen, Germany) equipped with gradient coil system capable of producing pulse gradient of up to 40 gauss/cm in each of the three directions. All MR images had been acquired with a parallel rat head surface coil (Bruker) and transmitter linear coil (Bruker). T_2_-weighted images were acquired at the beginning of each imaging session for accurate positioning of the animal inside the magnet bore. The T_2_-weighted images were acquired using the rapid acquisition with relaxation enhancement (RARE) imaging sequence with the following parameters: a repetition delay of 3000 ms, echo time of 44 ms, RARE factor of 8, and matrix dimension of 256 × 256 and two averages. 17 continuous slices with slice thickness of 1.2 mm were acquired with a FOV of 4.0 × 4.0 cm^2^. The total acquisition time per image in that series was 1 min 36 s. The T_2_-weighted MR images were acquired before the saline or OxAc/rGOT injection and also 70 min post injection.

### 3.6. Magnetic Resonance Spectrocopy (MRS)

Voxel shimming was performed by the Fastmap Protocol (Bruker). The field homogeneity typically resulted in signal line widths of 11 to 16 Hz for water. Water signal was suppressed by variable power radio frequency pulses with optimized relaxation delays (VAPOR). *In vivo* 1H MR Spectra of the brain were acquired by using a stimulated echo acuistion mode (STEAM) sequence with echo time = 3 ms, mixing time = 3 ms, repetition time = 2500 ms, 176 averages, cubic voxel size of 4 × 4 × 4 mm^3^. The total acquisition time per spectrum in that series was 5 min 55 s. BGS or saline administrations were administered into the tail vain without taking the rat out of the magnet bore. Baseline spectra were acquired 50 min before and 150 min after the injection of GOT or saline.

### 3.7. Data Analysis

All the MRS acquisition and analysis were performed without previous knowledge of the type of treatments. Selected spectral peak areas were obtained using TopSpin software (Bruker Biospin, Ettlingen, Germany). Areas (integrals) were measured between 3.10 and 2.80 ppm (PCr), 2.35 and 2.15 ppm (Glu), 2.15 and 1.80 ppm (*N*-acetylaspartate, NAA), 1.50 and 1.20 ppm (Lac) and 0.20 and 1.10 ppm (Lipid) for each spectrum. Despite often overlapping peaks and uneven baseline, the integrals were defined at exactly the same points in each phase spectrum. For the quantitative analysis, glutamate signals were normalized to creatine peak areas for each single spectrum. Percentage changes in the concentration of metabolites before and after injection was calculated for each time point for each rat, and two-sample *t*-tests were performed to assess the difference between the two groups.

## 4. Conclusions

To date, organophosphate-induced brain damage is an irreversible neuronal injury due to the fact that no pharmacological treatment is currently available to prevent or block secondary damage processes [19,20]. The major contributors to the secondary neuronal brain damage, manifested in cell death, are thought to be calcium influx and apoptosis as a result of excessive release of extracellular Glu [21]. It has been previously shown that following organophosphates intoxication there is a release of excess Glu in the brain, which appears to cause the neurological damage [3].

The current study confirmed the mechanism of BGS as an agent that accelerates the natural process of brain to blood glutamate efflux, following PO intoxication. In this article, for the first time, we have demonstrated real-time prolonged reduction of the excessive brain glutamate levels following BGS administration. This data provided the proof of concept and demonstrated the effectiveness of BGS as a neuroprotective treatment in the PO intoxication model. The fact that the intravenous administration of OxAc/rGOT significantly decreased the brain glutamate levels and kept it at a low level for the next few hours, strongly supports our previous findings in the PO intoxication model where we demonstrated that the BGS treatment is an effective neuroprotective agent and showed the blood glutamate reduction following BGS administration [13]. Our results showed that the BGS treatment significantly prevented the peripheral benzodiazepine receptor (PBR) density elevation, after PO exposure and was able to protect neurons in the piriform cortex of the treated rats.

In the current study, our MRS data shows that brain metabolic changes caused by PO intoxication can be seen already after 1 h. Our unexpected finding that following the BGS treatment there was also a significant decrease in brain lactate levels is very interesting. The determination of tissue hypoxemia in common medical practice is quite difficult. Lactate levels, which are not evident in normal brain tissue, are thought to indicate the presence of anaerobic metabolism [22] and the release of lactate is usually considered as an indication of cellular damage. Its significant decrease following BGS treatment suggests that this is perhaps an additional manifestation of the neuroprotection afforded by the lowering of brain glutamate levels.

In the last decade, many efforts have been made to develop a new neuroprotective treatment. NMDA antagonists or α-amino-3-hydroxy-5-methyl-4-isoxazolepropionic acid (AMPA) antagonists were among the most promising effective drugs. In experimental animal studies with soman poisoning, NMDA antagonists, such as MK-801 (Dizocilpine) and GK-11 (Gacyclidine), showed promising beneficial effects [5,23]. Unfortunately, in human clinical trials these drugs caused severe adverse effects and have not been approved for use [24].

We believe that BGS could serve as a candidate for a novel neuroprotective treatment in all pathological conditions in which high glutamate levels accumulate in the central nervous system and is one of the main causes for the neurological damage [4]. Intravenous treatment with recombinant GOT together with low amounts of oxaloacetate can be administered in combination with other established treatments since BGS is unlikely to have unwanted pathological consequences as its activity is only in the blood circulation. Humans have shown significantly differing natural GOT levels of between 7–45 U/L, inferring that the temporary augmentation of serum GOT levels should also not pose a problem. In addition, as there are many medical indications—e.g., liver diseases—in which levels of GOT are dramatically increased without overt symptoms, as has been shown in the current study, MRS is a very useful, non-invasive technology for the monitoring of brain glutamate and lactate levels following PO intoxication and could be used as a powerful and accurate tool in further pre-clinical and clinical trials.
